# A machine learning model that classifies breast cancer pathologic complete response on MRI post-neoadjuvant chemotherapy

**DOI:** 10.1186/s13058-020-01291-w

**Published:** 2020-05-28

**Authors:** Elizabeth J. Sutton, Natsuko Onishi, Duc A. Fehr, Brittany Z. Dashevsky, Meredith Sadinski, Katja Pinker, Danny F. Martinez, Edi Brogi, Lior Braunstein, Pedram Razavi, Mahmoud El-Tamer, Virgilio Sacchini, Joseph O. Deasy, Elizabeth A. Morris, Harini Veeraraghavan

**Affiliations:** 1 Department of Radiology, Memorial Sloan Kettering Cancer Center, New York, NY USA; 2 Department of Medical Physics, Memorial Sloan Kettering Cancer Center, New York, NY USA; 3 Department of Pathology, Memorial Sloan Kettering Cancer Center, New York, NY USA; 4 Department of Radiation Oncology, Memorial Sloan Kettering Cancer Center, New York, NY USA; 5 Department of Medicine, Memorial Sloan Kettering Cancer Center, New York, NY USA; 6 Department of Surgery, Memorial Sloan Kettering Cancer Center, New York, NY USA

**Keywords:** Breast cancer, Neoadjuvant chemotherapy, MRI, Radiomics, Machine learning

## Abstract

**Background:**

For breast cancer patients undergoing neoadjuvant chemotherapy (NAC), pathologic complete response (pCR; no invasive or in situ) cannot be assessed non-invasively so all patients undergo surgery. The aim of our study was to develop and validate a radiomics classifier that classifies breast cancer pCR post-NAC on MRI prior to surgery.

**Methods:**

This retrospective study included women treated with NAC for breast cancer from 2014 to 2016 with (1) pre- and post-NAC breast MRI and (2) post-NAC surgical pathology report assessing response. Automated radiomics analysis of pre- and post-NAC breast MRI involved image segmentation, radiomics feature extraction, feature pre-filtering, and classifier building through recursive feature elimination random forest (RFE-RF) machine learning. The RFE-RF classifier was trained with nested five-fold cross-validation using (a) radiomics only (model 1) and (b) radiomics and molecular subtype (model 2). Class imbalance was addressed using the synthetic minority oversampling technique.

**Results:**

Two hundred seventy-three women with 278 invasive breast cancers were included; the training set consisted of 222 cancers (61 pCR, 161 no-pCR; mean age 51.8 years, SD 11.8), and the independent test set consisted of 56 cancers (13 pCR, 43 no-pCR; mean age 51.3 years, SD 11.8). There was no significant difference in pCR or molecular subtype between the training and test sets. Model 1 achieved a cross-validation AUROC of 0.72 (95% CI 0.64, 0.79) and a similarly accurate (*P* = 0.1) AUROC of 0.83 (95% CI 0.71, 0.94) in both the training and test sets. Model 2 achieved a cross-validation AUROC of 0.80 (95% CI 0.72, 0.87) and a similar (*P* = 0.9) AUROC of 0.78 (95% CI 0.62, 0.94) in both the training and test sets.

**Conclusions:**

This study validated a radiomics classifier combining radiomics with molecular subtypes that accurately classifies pCR on MRI post-NAC.

## Background

The management of operable breast cancer has traditionally employed a tri-modality approach: surgery followed by adjuvant chemotherapy and radiation therapy. Beginning in 2001, randomized clinical trials have demonstrated the equivalency of neoadjuvant to adjuvant chemotherapy, findings of which have been practice-changing. Neoadjuvant chemotherapy (NAC) is given before surgery and has the advantage of allowing treatment monitoring and downstaging breast cancer, thereby decreasing the extent of local surgery [1, 2]. Its clinical implementation has allowed breast-conserving surgery (lumpectomy) and sentinel lymph node biopsy for women who historically required mastectomy and full axillary lymph node dissection [1, 2]. The goal of NAC is a pathologic complete response (pCR), defined as no remaining cancer in the breast. A pCR is a surrogate endpoint for improved disease-free and overall survival [3]. While surgery is currently required to confirm a pCR post-NAC, surgery may be obviated if pCR could be identified non-invasively.

Breast MRI is currently recommended pre- and post-NAC because it is the most accurate test for diagnosing a pCR compared with physical examination, mammography, and ultrasound [1, 4, 5]. However, the reported sensitivity of MRI for a pCR is quite variable; a meta-analysis reported a pooled sensitivity of 64% [6] which is not sufficient to obviate tissue confirmation and surgery [7]. High-resolution breast MRI holds a wealth of information that when combined with machine learning techniques has the potential to result in highly accurate and non-invasive NAC response detection methods. The field of radiomics involves the application of computer-automated quantitative analysis of images, augmenting visual assessment by extracting features not perceptible to human eye. These methods are amenable to integration with machine learning and have shown potential for non-invasive identification of treatment response in breast and other cancers [8–11]. Thus, the aim of our study was to develop and validate a radiomics biomarker that classifies breast cancer pCR post-NAC on MRI.

## Methods

### Patients

This retrospective Health Insurance Portability and Accountability Act-compliant study received Institutional Review Board approval, and written informed consent was waived. We identified consecutive women 18 years old or older with operable invasive carcinoma treated with NAC from 2014 to 2016 and who had (1) pre- and post-NAC breast MRI performed at our institution and (2) a post-NAC surgical pathology report assessing response. Exclusion criteria were (1) prior history of treated breast cancer, (2) breast MRI performed at an outside facility, (3) second primary cancer treated with chemotherapy, and (4) metastatic disease.

In total, 278 cancers from 273 patients (*n* = 5 bilateral synchronous invasive breast cancer) met the inclusion and exclusion criteria. There is no patient overlap in any prior published studies as well as in work currently undergoing review or in press. To ensure better generalization performance, we divided the dataset into training and testing sets (80/20 split). The training dataset consisted of 222 cancers (61 pCR, 161 no-pCR; mean age 51.8 years). The testing dataset consisted of 56 cancers (13 pCR, 43 no-pCR; mean age 51.3 years).

Clinical and pathologic data were extracted from the electronic medical record and included age, pre- and post-NAC pathology, NAC regimen, and dates of the pre- and post-NAC MRI examinations.

### MRI acquisition

All patients underwent a pre- and post-NAC contrast-enhanced MRI on a 1.5 or 3.0 Tesla system (Discovery 750, GE Medical Systems, Waukesha, WI) with a dedicated 8- or 16-channel breast coil (see Additional files 1 and 2 for protocol details). Axial T1-weighted fat-suppressed images were acquired pre- and post-contrast (continuously at three time points i.e., post-CE1, post-CE2, and post-CE3). The gadolinium-based contrast agent was administered at a concentration of 0.1 mmol gadobutrol per kg body weight (Gadavist; Bayer Healthcare Pharmaceuticals Inc., Whippany, NJ) at a rate of 2 ml/s. The acquisition parameters for conventional steady-state DCE-MRI were as follows: TR/TE = 7.9/4.3, flip angle = 12° in-plane spatial resolution = 1.1 × 1.1 mm, slice thickness = 1.1 mm, temporal resolution = ~ 120 s, axial orientation.

### Radiomics analysis

**Fig. 1:**
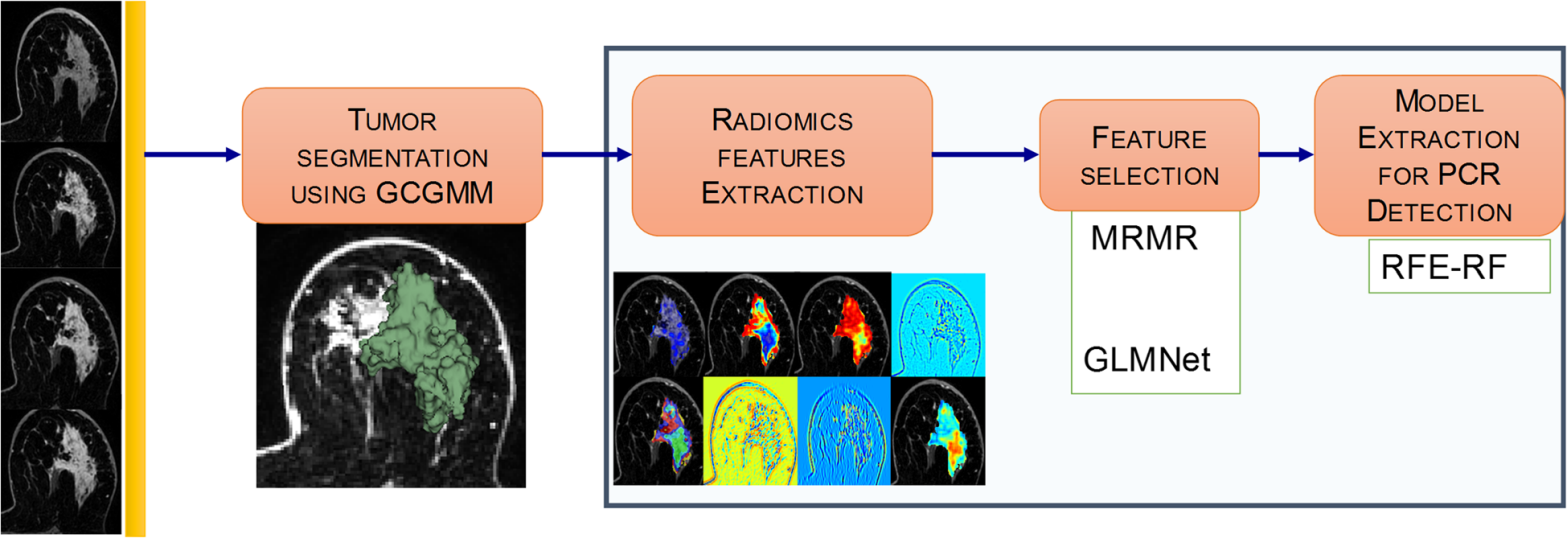
Framework for radiomics analysis. The Grow Cut Gaussian Mixture Model was used to generate volumetric tumor segmentation from the T1w DCE-MRI. Next, radiomics analysis was performed to extract the texture measures from the segmented volumes followed by machine learning analysis consisting of feature pre-filtering using Maximum Relevance Minimum Redundancy (MRMR) and generalized linear regression with elastic net constraints feature selection (GLMNet), followed by a recursive feature elimination random forest (RFE-RF) classifier for extracting a model for detecting a pCR

Figure 1 shows the framework for radiomics analysis. As shown, a single volumetric segmentation is generated for all the pre-contrast and three post-contrast MRIs separately for the pre-NAC and post-NAC MRIs. Scalar features summarizing features from within the segmented volumes including first-order histogram features, second-order Haralick texture features, Gabor edge features, and Haralick texture measures on Gabor edge maps were computed. The delta features were then computed by differencing the features on the post-NAC from the pre-NAC MRI features. A two-step explicit feature selection consisting of maximum relevance minimum redundancy (MRMR) followed by generalized linear regression using elastic net constraints was performed to pre-select the most relevant features. These features were then used in a recursive feature elimination random forest (RFE-RF) classifier with five-fold cross-validation to extract a model for classifying pCR.

#### Tumor segmentation

**Fig. 2:**
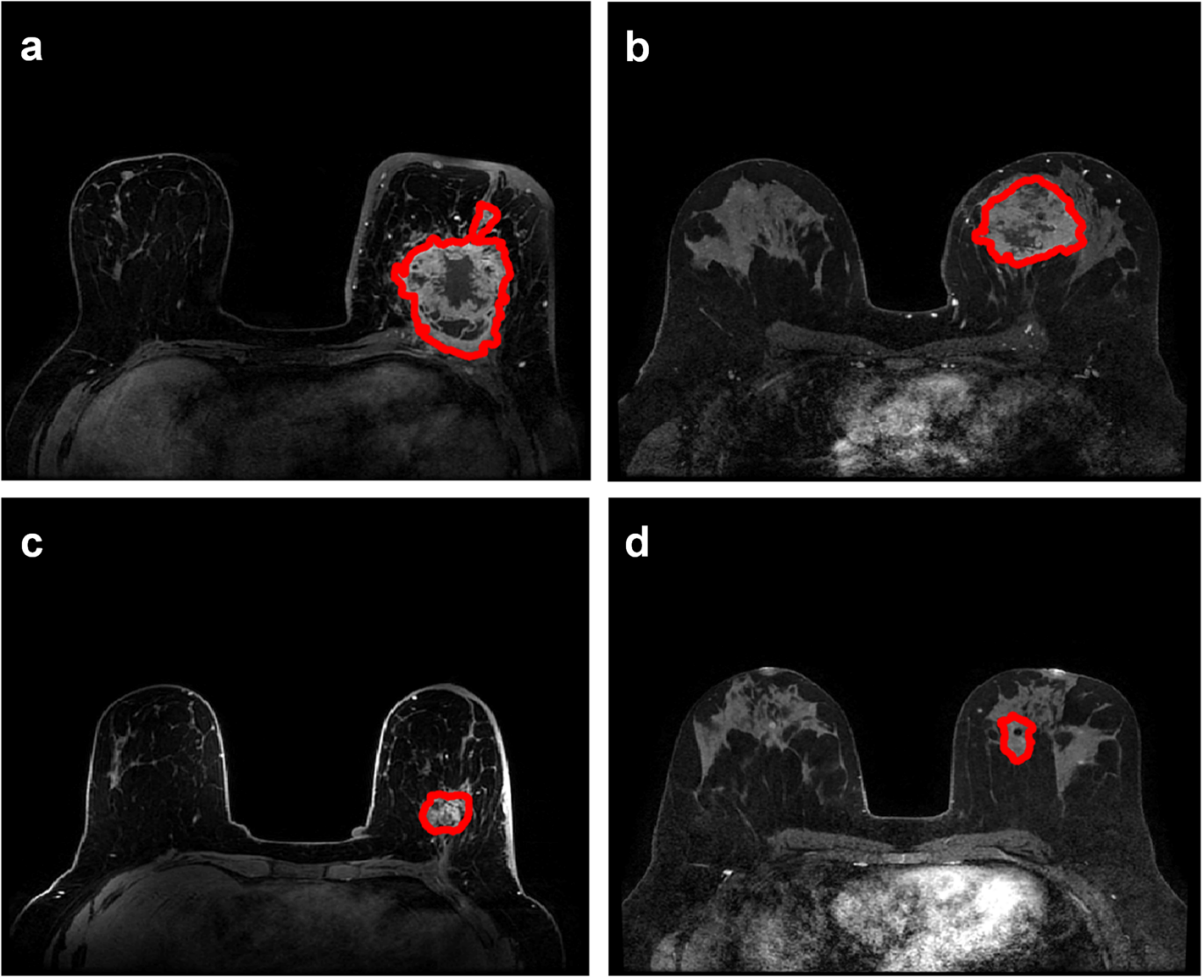
Representative pre-neoadjuvant chemotherapy (NAC) fat-saturated first post-contrast MRI (**a** and **b**) and post-NAC fat-saturated first post-contrast MRI demonstrating **c** no pathologic complete response (no pCR) for **a** and **d** pCR for **b**

Two breast imaging fellowship trained radiologists (EJS and NO, with 6 and 6 years of experience, respectively) performed single slice segmentation of the index breast cancer on the post-CE1 sequence. Figure 2 shows representative breast MRI pre- and post-NAC. If there was no visible tumor, the radiologists segmented the tumor bed based on the presence of tumor bed fibrosis, location of an accurately positioned pre-NAC biopsy marker, and/or anatomic landmarks. Concretely, the radiologists produced a conservative estimation of the enhancing lesion on the roughly the central slice containing the tumor. This segmentation was then used by the algorithm to compute a model of foreground tumor and the background regions.

The extracted model was then applied to all remaining slices in the image to extract a volumetric segmentation of the tumor. Tumors were volumetrically segmented using a previously developed in-house algorithm implemented in MATLAB (R2015b), the Grow Cut Gaussian Mixture Model [12]. Briefly, this algorithm computes a statistical model of the tumor appearance from the single slice segmentation and extends it to multi-slice volumetric segmentation. For our study, multi-phase contrast-enhanced T1-weighted fat-suppressed MRI consisting of pre- and three post-contrast images (four sequences per exam) were volumetrically segmented and the images were used to compute three temporal difference images (extracted by differencing the post-contrast images from pre-contrast MRI) and a voxel-wise tensor representation called the trace image (computed to integrate the images from the multi-phase MRI). Concretely, the pre- and three post-contrast image intensity values at each voxel were represented as a covariance matrix, with the diagonal entries corresponding to the squared values of the pre- and the three post-contrast signal intensities and the off-diagonal entries corresponding to the squared difference between these intensities. A voxel-wise tensor was then computed from this matrix through eigendecomposition. A trace value was computed from the top three eigenvalues of this decomposition at each voxel, from which a trace image was computed. This method produced a single segmentation for all of pre-contrast and the three post-contrast MRI images. The Grow Cut Gaussian Mixture Model-generated segmentations for all tumors were visually validated and corrected by one of the radiologists (EJS).

#### Radiomics feature extraction

All images were subjected to MRI histogram standardization [13] to harmonize the MRI images prior to feature extraction as was done in previously in Um et al. [14]. We extracted a total of 255 radiomics features for each breast cancer. We used the same four sequences (T1-weighted fat-saturated pre-contrast and three post-contrast sequences) from the pre- and post-NAC MRI (total of 8 sequences). Twenty-one radiomics features were computed for each sequence and consisted of (a) first-order histogram features consisting of the mean, standard deviation, kurtosis, and skewness (*m* = 4); (b) second-order Haralick texture features [15] consisting of energy, entropy, correlation, contrast, and homogeneity (*m* = 5); (c) features from Gabor edge maps [16] extracted using a bandwidth of *γ* = 1.414 and at angles *θ* = {0°, 90°} (*m* = 2); and (d) Haralick texture measures computed from Gabor edge maps (*m* = 10). In addition, one intra-tumor cluster entropy measure (*n* = 1) was computed for the pre- and post-NAC MRI (see Additional file 1). The intra-tumor cluster entropy quantified the textural variability within sub-regions in the tumor. The sub-regions represent regions within the tumor that have similar textural values. These clusters were automatically computed using self-tuning spectral clustering using five Haralick textures as described in detail in Additional file 1. This resulted in 85 features for the pre- and post-NAC MRI for a total of 170 features. The individual features were scalar (or single) values that summarized the whole segmented volume. Delta texture measures were then computed to evaluate change by subtracting the individual scalar features from the four post-NAC MRI sequences and the corresponding pre-NAC MRI (*m* = 85) resulting in a total of 255 radiomics features per patient.

Of note, only five Haralick textures were computed instead of 13 because these features are known to be correlated with each other [17] and have been shown to be useful for radiomics classification in prior works including [18, 19]. Cluster shade and prominence features were removed as these are more useful for clustering and segmentation instead of capturing variability within volumes. We computed Gabor edge features only at two angles and a fixed bandwidth to reduce the number of correlated features and the chances of producing an over-optimistic classifier.

#### Feature selection and machine learning for radiomics-based response assessment

Feature pre-selection methods were pre-decided prior to performing the analysis. Sequential MRI radiomics feature selection was performed using (i) MRMR [20] and (ii) a generalized linear regression model [21] using elastic net constraints. MRMR ranked the features according to their relevance with the goal of reducing redundancy that occurs when using highly correlated features. The relevant features from MRMR ranking were further reduced using elastic net regression. The regression analysis was applied with elastic net constraints using three-fold cross-validation. A model of the outcome variable (pCR) was computed. The set of variables (or features) with an importance > 0.1 in this model were selected and used as inputs for the RFE-RF classifier [22]. Feature pre-selection using MRMR followed by elastic net regression and recursive feature elimination was done for handling correlated features and to reduce the chances of highly over-optimistic classification and that would lead to the best generalization performance on an independent test set. Class imbalance between the minority (pCR) and majority class (not pCR) was resolved using the synthetic minority oversampling technique (SMOTE) as has been used for machine learning MRI radiomics-based classifiers [18, 19]. Using SMOTE, additional samples were generated with higher likelihood (3:1) for the minority class compared to the majority class. The new samples were generated by averaging *K* = 5 nearest neighbors of a particular class with the samples chosen randomly. Prior to SMOTE resampling, all radiomics features were scaled and centered using *z*-score standardization. SMOTE-based augmented samples were generated only for the training set. We fixed the parameter for selecting the features from MRMR (top 80%), and hyper-parameter selection for elastic net regression was done using cross-validation.

Starting with 255 features, MRMR reduced the number of features to 204. The elastic net regression method reduced these features further to 156. In the case of model 3 that was trained with 243 features after removing all the MRI intensity features, the MRMR reduced the features to 204, while the elastic net reduced the features to 51 features.

RFE-RF machine learning [23] was subsequently trained with repeated and nested five-fold cross-validation using 1000 trees. The RFE-RF classifier conducted additional feature selection using explicit and implicit methods. The recursive feature elimination method performed explicit feature selection by ranking features that have a maximum impact on accuracy upon their removal. The random forest method performed implicit feature selection by selecting a random set of features in each node of the classification tree, which were then evaluated for splitting the data into pCR versus no CR classes. Features were selected in each tree node that successively increased the chances of accurate classification. Additional details are in Additional file 1. Two radiomics RFE-RF classifier models were constructed, one including only the radiomics features (model 1) and the second also including the breast cancer molecular subtype (model 2). The molecular subtype was treated as a categorical variable in the classifier and included in the RFE-RF classifier. In addition, we evaluated the performance of radiomics classifier without MRI intensity features (model 3) to assess the utility of MRI radiomics measures without the MRI intensity metrics. The results of this model are included in Additional file 2, Supplemental Table 4.

### Statistical analysis

All machine learning analysis was performed with nested cross-validation with data set aside for independent testing using R (version 3.3) software [24]. The accuracy of each RFE-RF classifier model was assessed using area under the receiver operating characteristic curve (AUC), true positive, true negative, false positive, false negative rates, positive predictive value, and negative predictive value. RFE-RF classifiers computed using model 1 and model 2 as well as model 1 vs. model 3 were compared by evaluating the differences between the receiver operating curves using the *ROC.test* method available in the pROC package [24]. Statistical associations of radiomics features found to be most relevant using the RFE-RF classifiers with pCR were assessed using the two-sided Wilcoxon test. Statistical corrections for multiple comparisons were performed using the Benjamini Hochberg correction. Only *P* values < 0.05 were considered significant. The packages pROC, rms, caret, and mRMRe in the R Core Team (version 3.3) software [24] were used for statistical analysis. All analysis was performed after the image features were standardized using *z*-score standardization to centralize all the features to zero mean Gaussian distribution.

### Availability of code

The radiomics features were extracted using the open-source software library CERR software available through (https://github.com/cerr/CERR) [25]. The methods for computing the machine learning classification models are available through the author’s Github link (https://github.com/harveerar/Code).

## Results

### Clinical characteristics

Patient characteristics in the training and testing cohorts are given in Table 1. Histologic confirmation was available for the entire study population. From a total of 283 segmented cancers, we excluded patients who had (a) technically inadequate post-NAC MRI for analysis (*n* = 1), (b) no information on molecular subtype (*n* = 1), and (c) biopsy-proven metastatic disease during NAC (*n* = 3). The final cohort consisted of 278 cancers from 273 patients as 5 patients had bilateral synchronous breast cancer.

**Table 1:** Characteristics of patients in the training and testing sets. *P* values correspond to measures computed using Wilcoxon rank-sum tests performed to compare the training and testing cohorts

**Characteristics**	**Cohort**		
	**Training**	**Testing**	***P*value**
*n*	**222**	**56**	
Age, mean ± SD, years	51.8 (11.8)	51.3 (11.8)	0.90
*Pathologic response* (*%*)			0.50
pCR	61 (27.5)	13 (23.2)	
no-pCR	161 (72.5)	43 (76.8)	
*Histology*, *no* (*%*)			0.90
Invasive ductal	203 (91.4)	51 (91.1)	
Invasive lobular	8 (3.6)	3 (5.4)	
Mix	5 (2.3)	0 (0)	
Invasive NOS	6 (2.7)	2 (3.5)	
*Molecular subtype*, *no* (*%*)			0.40
HR+HER2−	76 (34.2)	22 (39.3)	
HR+HER2+	52 (23.4)	9 (16.1)	
HR−HER2+	36 (16.2)	8 (14.3)	
Triple negative	58 (26.2)	17 (30.3)	

*Abbreviations*: *pCR* pathologic complete response, (*+*) positive, (*−*) negative; *NOS *not otherwise specified

*P value < 0.05

There was no significant difference between the training and testing sets regarding the prevalence of pCR: 61/222 (27.5%) of patients and 13/56 (23%) of patients had a pCR in the training and testing sets, respectively (*P* = 0.50). There was no significant difference between the two sets regarding breast cancer molecular subtype (*P* = 0.40).

### Performance of RFE-RF classifier models for detecting pCR on training and test sets

**Fig. 3:**
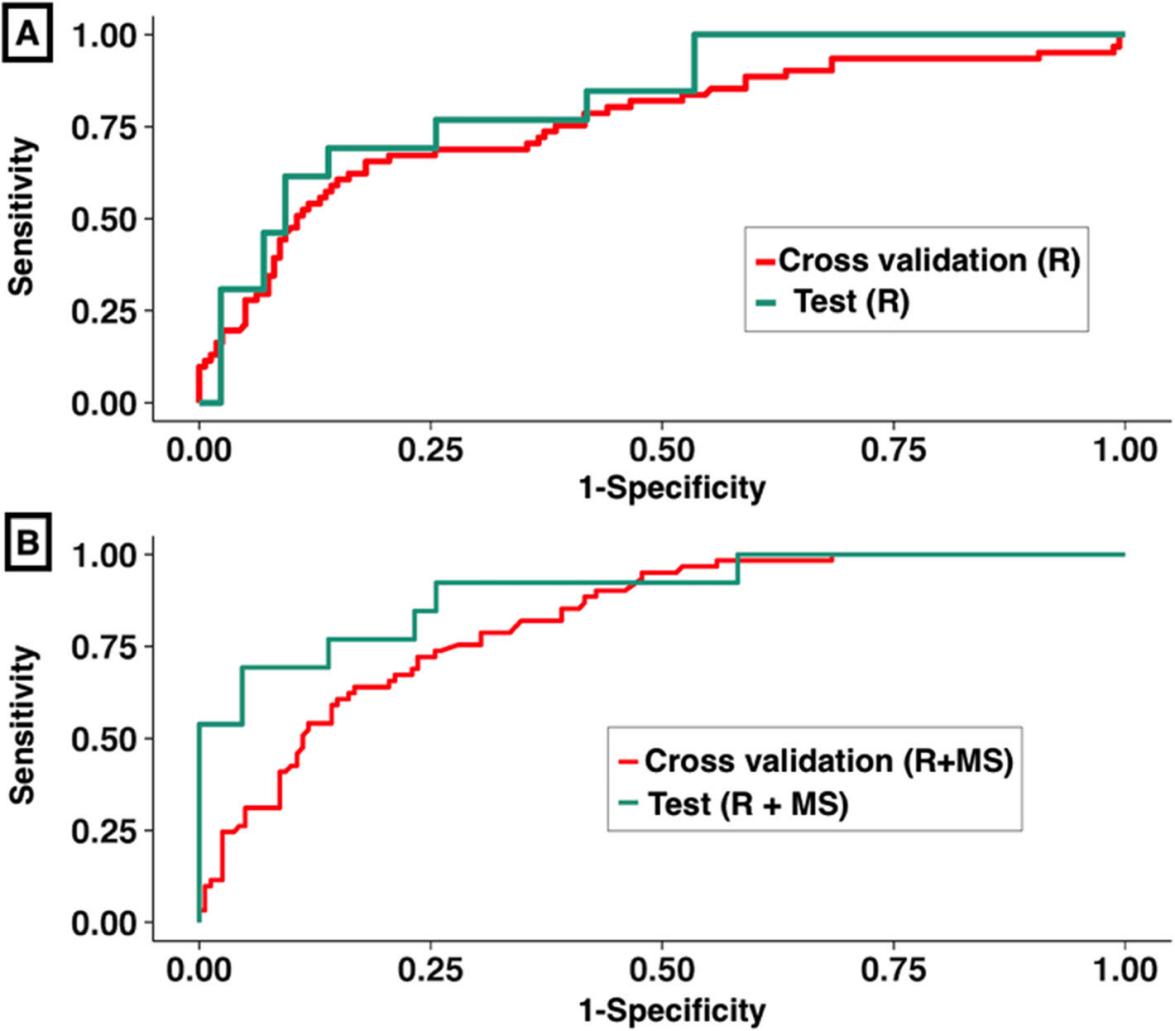
Receiver operating curves (ROC) for **a** radiomics (i.e., R) and **b** radiomics with molecular subtype (i.e., R+MS) classifier models in the training and testing sets. Repeated five-fold, nested cross-validation was performed in the training set wherein the accuracy values for the classifier were produced by evaluating the classifier only on the data not used in the model building in each fold of the validation. An independent hold-out set that was never seen by the model during training was treated as the test set

Table 2 shows the achieved accuracies of the RFE-RF classifier models. Model 1 achieved a slightly lower but not significantly different AUROC on cross-validation of 0.72 (95% CI 0.64, 0.79) in the training set and similarly accurate (*P* = 0.10) AUROC of 0.83 (95% CI 0.71, 0.94) in the test set. In comparison, model 2 achieved a cross-validation AUROC of 0.80 (95% CI 0.72, 0.87) in the training set and a similarly accurate (*P* = 0.90) AUROC of 0.78 (95% CI 0.62, 0.94) in the test set. Model 3 that was trained without any of the MRI intensity features and only the radiomics features achieved a similarly accurate classification as model 1 with a cross-validation AUROC of 0.71 (95% CI 0.64, 0.79) with (*P* = 1.0) and testing AUROC of 0.78 (95% CI 0.62, 0.94) with (*P* = 0.4). Figure 3 shows the ROCs that were computed using model 1 and model 2 on both the cross-validation and testing cohorts. Model 1 produced highly similar accuracies on the training and testing cohorts. A similar trend was observed for model 2, with no significant difference in the training and testing results. We benchmarked the performance of these two models to other classifiers (see Additional file 1).

**Table 2:** Performance of the RFE-RF classifier trained using model 1 and model 2 for predicting a pCR. *P* values are derived from comparison of the ROC curves computed for the cross-validation and test sets for model 1 and model 2

	*Model 1*		*Model 2*	
	**Radiomics**		**Radiomics and molecular subtype**	
	***Training***	***Testing***	***Training***	***Testing***
AUROC 95% CI	0.72 (0.64, 0.79)	0.83 (0.71, 0.94)	0.80 (0.72, 0.87)	0.78 (0.62, 0.94)
Sensitivity or TPR (no-pCR)	0.73 (0.65, 0.79)	0.77 (0.61, 0.88)	0.78 (0.70, 0.84)	0.79 (0.64, 0.90)
Specificity or TNR (pCR)	0.64 (0.51, 0.76)	0.69 (0.39, 0.91)	0.69 (0.56, 0.80)	0.69 (0.39, 0.91)
PPV	0.84 (0.77, 0.90)	0.89 (0.75, 0.97)	0.87 (0.80, 0.92)	0.89 (0.75, 0.97)
NPV	0.47 (0.36, 0.58)	0.47 (0.24, 0.71)	0.54 (0.42, 0.65)	0.50 (0.26, 0.74)
	*P* value = 0.1		*P* value = 0.9	

*Abbreviations*: *AUC* area under the receiver operating characteristic curve, *pCR* pathologic complete response, *TPR* true positive rate, *TNR* true negative rate

*P value < 0.05

**Fig. 4:**
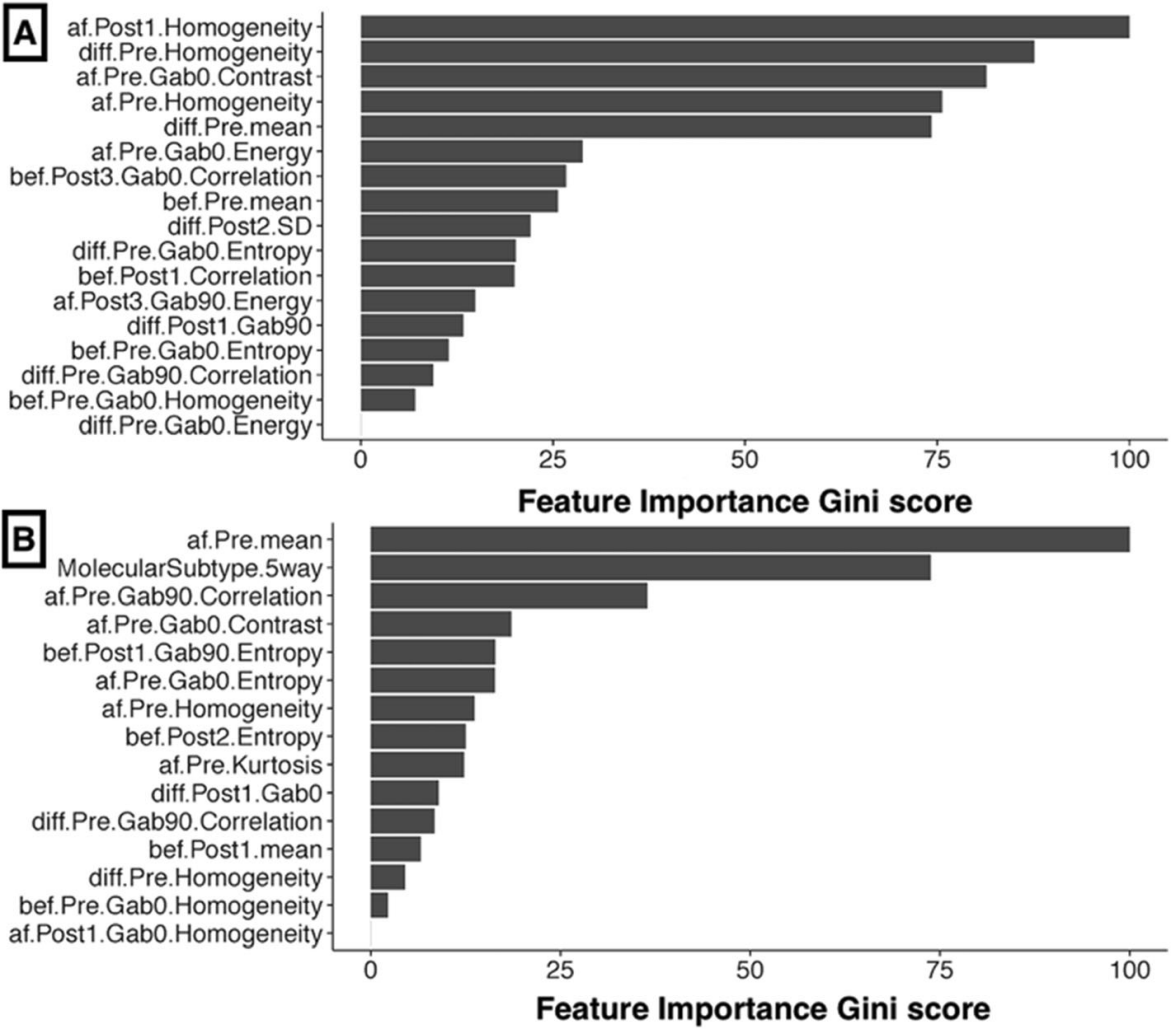
Relative importance of the radiomics features and molecular subtype as selected by the recursive feature elimination random forest (RFE-RF) classifier for the **a** radiomics only model and **b** radiomics with molecular subtype model. “af” corresponds to post-neoadjuvant chemotherapy (NAC), “bef” to pre-NAC, and “diff” to difference between post-NAC and pre-NAC radiomics features. “Pre” corresponds to pre-contrast MRI, post1 to the first-post contrast, post2 to the second post-contrast, and post3 to the third post-contrast of the multi-phase DCE-MRI sequence. Gab0 corresponds to Gabor edge feature computed at 0°, while Gab90 to the Gabor edge feature computed at 90°. A bandwidth of 1.414 was chosen for the Gabor textures for all orientations. Feature importance corresponds to the Gini importance measure used by the random forest model

Figure 4 shows the order of feature importance by their relevance for classification as determined by the RFE-RF classifier for model 1 and model 2. Feature importance corresponded to the Gini importance measure used to rank features in the RF classifier [26]. The model 1 RFE-RF classifier identified 19 different features including pre-contrast and first post-contrast MRI intensity features from post-NAC and difference from the post-NAC to pre-NAC mean intensities, as well as multiple post-NAC features such as post-NAC first post-MRI Gabor (90, 1.414) entropy, post-NAC pre-contrast MRI contrast texture, and delta radiomics features such as first post-contrast MRI Gabor (0, 1.414) energy, and delta pre-contrast contrast textures. These features were found to be significantly different between pCR and no CR when evaluated on the entire dataset combining training and testing sets (see Additional file 2, Supplemental Table 2). Model 2 identified 12 radiomics features and molecular subtype as relevant for pCR classification. Of these 12, delta pre-contrast mean MRI intensity, delta pre-contrast MRI homogeneity, delta second post-contrast MRI standard deviation, and first post-contrast MRI mean intensity were all significantly different between pCR and no CR on the whole dataset (see Additional file 2, Supplemental Table 3). Model 3 identified 11 radiomics features, of which delta pre-contrast MRI homogeneity, delta pre-contrast MRI contrast, and delta first post-contrast MRI Gabor (90, 14.14) energy were significantly different between pCR and no CR (see Additional file 2, Supplemental Table 5).

## Discussion

We developed and validated a combined radiomics and molecular subtype-based classifier model for assessing pathologic complete response (pCR) with high accuracy and reproducibility. Our study combines pre-NAC and post-NAC MRI to compute delta radiomics features for classifying pCR from DCE-MRI images. This non-invasive image-based radiomics marker could assist the radiologist and improve diagnostic accuracy while facilitating the standardization of post-NAC MRI reporting. This is important because the current criteria for the evaluation of tumor response, such as those defined by the World Health Organization [27] and the response evaluation criteria in solid tumors (RECIST) by the European Organization for Research and Treatment of Cancer [28], are not used to identify a pCR as they do not include pertinent criteria specific to breast imaging [29]. Furthermore, routine breast imaging is not sufficiently accurate for identifying a pCR to replace surgery [29]. A meta-analysis demonstrated that MRI is more sensitive than mammography and ultrasound in identifying a pCR. However, the accuracy of MRI when using no enhancement of the tumor bed on post-NAC MRI had a specificity of 0.54 (95% CI = 0.39–0.69), demonstrating the need for a better image-based method of response assessment [30].

Our findings are relevant for an increasing number of clinical trials challenging the status quo that all breast cancer patients must undergo surgery. Heil et al. [31], Rauch et al. [32], and Kuerer et al. [33, 34] previously showed the potential utility of image-guided biopsies as an alternative to surgery in exceptional responders to NAC. Thus, a few clinical trials are underway to study the omission of surgery (e.g., the national cooperative group multicenter feasibility trial NRG BR005 and NCT02945579 by MD Anderson) and a few are being planned (e.g., the multicenter trial [RESPONDER] in the USA, and NOSTRA and MICRA [35] in the UK and the Netherlands, respectively).

We evaluated radiomics features from both pre- and post-NAC MRI. Several studies have evaluated the utility of pre-NAC MRI to identify a pCR. For example, Braman et al. [36] evaluated the intra- and peritumoral radiomics features in 117 patients; the combined feature set yielded a maximum AUC of 0.78 ± 0.03, which improved with the inclusion of molecular subtype. Channing's et al. [37] performed texture analysis using 85 pre-NAC MRIs and found that kurtosis was associated with a pCR in non-triple-negative breast cancers. Fan et al. [38] found 12 radiomics features using 57 pre-NAC MRIs to be associated with tumor response to NAC. Wu et al. [39] found that imaging heterogeneity using a multiregional spatial interaction-based marker was independently associated with recurrence-free survival. Cain et al. [40] developed a multivariate machine-learning model using 288 pre-NAC MRIs and achieved an AUC of 0.707 in identifying pCR of triple-negative/HER2 breast cancers. Tahmassebi et al. [41] evaluated multiparametric pre-NAC MRI in 38 patients and reported an AUC of 0.86 to identify residual cancer burden class with zero being defined as a pCR.

Our accuracies are comparable to those from several studies that compared pre-NAC MRI and MRI at an early-treatment time point during NAC. Thibault et al. [42] conducted their analysis in 38 patients who underwent pre-NAC and MRI post-6–8 NAC cycles, extracting 1043 texture features; the best feature-map pair identified a pCR with 100% sensitivity and specificity. However, this was a small test cohort that was not independently validated. Drukker et al. [43] found in 127 patients with a pre- and early treatment MRI that the most-enhancing tumor volume predicted recurrence-free survival post-NAC. In our study, we found that the difference between post-NAC and pre-NAC in the mean intensity from pre-contrast T1-weighted fat-saturated MRI, as well as the post-NAC pre-contrast T1-weighted fat-saturated mean, was associated with pCR. Additional texture and edge-based measures including post-NAC Gabor edge-based textures, and delta radiomics measures such as contrast, Gabor edge-based energy, and standard deviation showed significant difference between pCR and nCR. We additionally evaluated the performance of the radiomics classifier after removing the MRI intensities to determine the contribution of the texture and edge-based metrics for pCR classification (model 3). We found that this model performed similarly as the radiomics classifier incorporating the MRI intensities, clearly indicating the utility of texture and edge-based radiomics measures in classifying pCR. These results show the value of including features from both pre-NAC and post-NAC MRI for assessing pCR.

We used an automated radiomics approach, which included a semi-automated volumetric tumor segmentation method, thus representing an improvement in accuracy over other models with similar number of patients. This segmentation method has been shown to produce more reproducible radiomics features and ultimately improve machine learning classifier accuracy compared with radiomics features computed from manually delineated volumes of interest (VOIs) and VOIs generated using two other frequently used segmentation methods [12].

We added breast cancer molecular subtype to our radiomics biomarker because molecular subtype determines the likelihood of pCR post-NAC [1]. The combination of radiomics features with molecular subtype resulted in a clear improvement in classification performance for detecting a pCR over that of molecular subtype alone and a moderate improvement over that of radiomics features alone. This suggests that radiomics features modeling heterogeneity in the imaging phenotype have the potential to add value to genomic signature-based predictors of response. Nevertheless, NAC response is known to vary even within a breast cancer molecular subtype, often due to genomic intra- and inter-tumor heterogeneity [44]. Genomic heterogeneity is a major factor in determining treatment response and cancer progression, and it is a driver of treatment resistance [45]. Therefore, further study with a larger dataset and combining such radiomics measures with additional genomic factors could help validate the potential for such integrated markers of response.

Our study has several limitations. First, this was a retrospective, single-institution analysis that was validated internally by an RFE-RF classifier but not validated externally. Second, MRI was performed using two different field strengths and two different breast coils. A third issue may stem from variability in segmentations that reduce the reproducibility of radiomics features. We mitigated this variability by using a semi-automated method that was shown to result in more reproducible radiomics measures than manual delineations. However, a fully automated method is desirable. Our group is working on a deep learning-based automated segmentation method to further improve radiomics feature reproducibility.

## Conclusions

In conclusion, we developed and validated a machine learning model combining radiomics with molecular subtypes that accurately predicts pCR on MRI with an AUROC of 0.88 in an independent test set. Our results highlight the potential clinical value of including radiomics-based feature classifiers to predict pCR post-NAC.

## Supplementary information

**Additional file 1.** Supplemental Narrative. Multiparametric MRI, Neoadjuvant Protocol, Additional Feature Selection by the RFE-RF Classifier, Performance of RFE-RC Classifier Models 1 and 2 Compared to Others, Intra-tumor Heterogeneity Feature.

**Additional file 2.** Supplemental Tables 1–5.

**Additional file 3.** Supplemental Figure.

## Data Availability

The datasets used and/or analyzed during the current study are available from the corresponding author on reasonable request.

## Abbreviations

AUROC: Area under the receiver operating characteristic curve
NAC: Neoadjuvant chemotherapy
pCR: Pathologic complete response pCR
RFE-RF: Recursive feature elimination random forest
MRMR: Maximum relevance minimum redundancy
SMOTE: Synthetic minority oversampling technique
