# Supplementary material for: A machine learning model that classifies breast cancer pathologic complete response on MRI post-neoadjuvant chemotherapy
Source: Breast Cancer Res. 2020 May 28;22:57. doi: 10.1186/s13058-020-01291-w (PMC7254668; doi:10.1186/s13058-020-01291-w)
Supplement: Supplementary file 1 — Additional file 1. Supplemental Narrative. Multiparametric MRI, Neoadjuvant Protocol, Additional Feature Selection by the RFE-RF Classifier, Performance of RFE-RC Classifier Models 1 and 2 Compared to Others, Intra-tumor Heterogeneity Feature. [file 13058_2020_1291_MOESM1_ESM.pdf]

ADDITIONAL FILE 1

Supplemental Narrative

Multiparametric MRI:

All patients undergoing NAC at our institution had a pre- and post-NAC standard-of-care clinical breast MRI. All post-NAC MRI take place after the last cycle Paclitaxel, which is approximately 16 weeks after the pre-NAC MRI. We leveraged these diagnostic studies (**Table in Additional File 2**). All MRI studies were performed on either a 1.5 or 3.0-Tesla GE Signa whole-body MRI unit (GE Medical Systems, Waukesha, WI) equipped with a dedicated 8 or 16-channel surface breast coil. All sequence parameters were similar. **The table in Additional File 2** illustrates the NAC Breast MRI protocol.

Neoadjuvant Protocol:

<u>HER2 Negative breast cancers:</u> All patients will be treated with the same standard of care systemic NAC regimen at MSK, AC-T (Doxorubicin-cyclophosphomide-paclitaxel). The MSK regimen is AC (Doxorubicin-cyclophosphomide) given every 2 weeks for four cycles; followed by Paclitaxel (T) given every 2 weeks for four cycles (surgery is performed after the last cycle of Paclitaxel).

<u>HER2 Positive breast cancers:</u> All patients will be treated with the same standard-of-care systemic NAC regimen at MSK, AC-THP (Doxorubicin-cyclophosphomide-paclitaxel-traztuzumab-pertuzumab). The MSK regimen is AC (Doxorubicin-cyclophosphomide) given every 2 weeks for four cycles; followed by Paclitaxel (T) given every 2 weeks for four cycles

(surgery is performed after the last cycle of Paclitaxel). HP (traztuzumab-pertuzumab) is started concurrently with Paclitaxel and given for 1 year.

Additional Feature Selection by the RFE-RF Classifier:

In the explicit method, the features were recursively ranked by importance using the RFE procedure. The RF is an ensemble classifier consisting of a number of decision tree classifiers that perform implicit feature selection by computing the relative importance of individual features using the Gini importance measure (1). The Gini measure was computed in each node split of the individual trees and efficiently captured how well the data could be split into the two classes at the individual nodes given the set of features selected to split the data into the individual groups at that node. The nodes refer to each decision point in each tree of the RF classifier (1). The hyper-parameters for the RF classifier, namely, the number of features selected for node splitting, were optimized in nested cross-validation.

Performance of RFE-RC Classifier Models 1 and 2 Compared to Others:

We compared the performance of Models 1 and 2 radiomics classifiers that use both the pre-NAC and post-NAC MRI to corresponding radiomics classifiers (1- radiomics only and 2- radiomics and molecular subtype) that use only the pre-NAC MRI. Both pre-NAC classifier models achieved low cross-validation accuracies using the training set: the pre-NAC radiomics only model achieved an AUROC of 0.54 (95% CI: 0.46, 0.63), sensitivity of 0.75 and specificity of 0.28, while the pre-NAC radiomics with molecular subtype model achieved an AUROC of

0.53 (95% CI: 0.44, 0.61), sensitivity of 0.63 and specificity of 0.31. They also achieved low accuracies using the test set: the pre-NAC radiomics only model achieved an AUROC of 0.66 (95% CI: 0.50, 0.82), sensitivity of 0.67 and specificity of 0.385 and the pre-NAC radiomics with molecular subtype model achieved an AUROC of 0.61 (95% CI: 0.45, 0.76), sensitivity of 0.67 and specificity of 0.385. None of the pre-NAC radiomic features selected to be relevant by either of the models was significantly associated with a pCR.

Finally, we evaluated the feasibility of using the molecular subtype alone to distinguish a pCR using ROC analysis. The cut-point maximizing the AUROC was determined using the Youden index with the training set and was applied to the test set. Molecular subtype only yielded an AUROC of 0.65 (95%: CI: 0.60, 0.70) in the training set and 0.65 (95% CI: 0.56, 0.75) in the test set, indicating that the radiomics-based classification outperformed the molecular subtype-based classification.

Intra-tumor Cluster Entropy Feature:

The intra-tumor cluster entropy feature is based on the inter-site tumor heterogeneity entropy feature used in our prior work (2) where the inter-site measures were computed between different sites of disease within the same patient. In this work, we computed the said feature to quantify the textural heterogeneity within sub-regions of the same tumor. The intra-tumor cluster entropy feature was extracted using the following steps.

First, sub-regions of uniform texture were extracted within each tumor. The sub-regions were computed by clustering voxel-wise textures using Gaussian mixture model clustering. The

appropriate number of clusters were automatically extracted by maximizing the Akaike information criterion (AIC). Self-tuning spectral clustering was computed by using the five Haralick texture features from pre-contrast and the three post-contrast images as a vector of features (m = 5 × 4 = 20 features).

Each sub-region within the tumor was summarized by the mean texture values of all the features. The textural differences between each pair of sub-regions were computed using Euclidean distance measure. The sub-region dissimilarities were then normalized to be within 0 and 1. The differences in the distribution of sub-region dissimilarities were summarized using the Shannon entropy formulation, where N is the number of sub-regions, d_n corresponds to the normalized dissimilarity between sub-region pairs, and $p(d_n)$ corresponds to the normalized frequency of that dissimilarity.

$$SE = -\frac{1}{N}\sum_{n=1}^{N} p(d_n) log_2(p(d_n))$$

References

1. Breiman LJML. Random Forests. 2001;45(1):5-32.

2. Vargas HA, Veeraraghavan H, Micco M, Nougaret S, Lakhman Y, Meier AA, et al. A novel representation of inter-site tumour heterogeneity from pre-treatment computed tomography textures classifies ovarian cancers by clinical outcome. European radiology. 2017;27(9):3991-4001.
