# Supplementary material for: A machine learning model that classifies breast cancer pathologic complete response on MRI post-neoadjuvant chemotherapy
Source: Breast Cancer Res. 2020 May 28;22:57. doi: 10.1186/s13058-020-01291-w (PMC7254668; doi:10.1186/s13058-020-01291-w)
Supplement: Supplementary file 2 — Additional file 2. Supplemental Tables 1–5. [file 13058_2020_1291_MOESM2_ESM.pdf]

ADDITIONAL FILE 2

Supplemental Table 1: NAC Breast MRI protocol used in all patients.

**Series Descriptor**	**T2-Weighted Fat-Saturated**	**T1-Weighted**	**Steady State DCE-MRI T1-Weighted Fat-Saturated**
**In-Plane Resolution (mm^2)**	1.0 × 1.0	1.0 × 1.0	1.0 × 1.0
**Slice (mm) Thickness**	3.0	1.0	1.0
**TE/TR (ms)**	102/5200	2.5/5.2	2.1/4.4
**Bandwidth (kHz)**	42	63	83
**NEX**	2	1	1
**Acquired Matrix**	224 × 228	300 × 300	300 × 300
**Acceleration Factor**	2.5	2	2.5
**Number of time points**	1	1	Pre-contrast + 3 post-contrast

Abbreviations: DCE-MRI, dynamic contrast-enhanced magnetic resonance imaging; NAC, neoadjuvant chemotherapy

Supplemental Table 2: Median and inter-quartile range (IQR) between pCR and nCR among all the features selected as relevant by RFE-RF classifier for the Radiomics only Model 1 classifier. Adjusted P values are shown. Actual values of the features are shown. Note that the classifier uses z-scale standardized features for placing uniform importance to features initially.

**Feature**	**pCR median (IQR)**	**nCR median (IQR)**	**P values adjusted**
Difference pre-contrast Mean	-0.10 (-0.44, 0.44)	0.30 (-0.03, 0.785)	**< 0.001**
Post-NAC pre-contrast Gabor(0, 1.414) mean	526 (322, 694)	467 (119, 752)	0.299
Post-NAC post1 Gabor(90, 1.414) entropy	-0.50 (-0.72, -0.13)	-0.32 (-0.67, 0.03)	**0.035**
Post-NAC post1 homogeneity	2091 (1389, 2720)	2325 (676, 3892)	0.969
Post-NAC post1 mean	14.0 (8.0, 19.0)	9.3 (5.5, 15.2)	**0.003**
Difference post2 skewness	6.2 (3.2, 11.6)	3.5 (0.30, 8.2)	**0.003**
Post-NAC post1 Gabor(90, 1.414) energy	2186 (1418, 2885)	2488 (692, 4097)	0.969
Post-NAC pre-contrast Gabor(0, 1.414) energy	68.0 (49.0, 76.0)	56.0 (38.0, 71.0)	0.491

Post-NAC pre-contrast Gabor(90, 1.414) entropy	1944 (1250, 2439)	2036 (599, 3541)	0.984
Difference post1 mean	6.1 (3.2, 11.6)	3.5 (0.30, 8.20)	**0.003**
Difference post1 Gabor (0, 1.1414) energy	0.09 (-0.22, 0.58)	0.47 (0.04, 0.88)	**0.003**
Difference pre-contrast contrast	6.1 (2.8, 11.4)	3.5 (0.34, 8.47)	**0.007**
Post-NAC pre-contrast contrast	13.0 (8.0, 18.0)	9.3 (5.5, 15.2)	**0.007**
Pre-NAC post2 homogeneity	6.8 (5.0, 9.7)	6.5 (4.3, 9.6)	0.392
Post-NAC post1 SD	72.0 (59.0, 90.0)	60.0 (43.0, 79.0)	**0.002**
Difference post1 Gabor (90, 1.414) contrast	-228 (-861, 256)	-50 (-540, 251)	0.206
Pre-NAC post2 contrast	32.0 (25.0, 42.0)	30.0 (23.0, 40.0)	0.589
Pre-NAC pre-contrast Gabor (90, 1.414) correlation	1155 (343, 1719)	983 (336, 1431)	0.299
Difference post1 Gabor (90, 1.1414) correlation	0.97 (-1.55, 5.10)	0.72 (-1.21, 3.00)	0.589

Supplemental Table 3: Median and inter-quartile range (IQR) between pCR and nCR among all the features selected as relevant by RFE-RF classifier for Model 2. Adjusted P values are shown. Actual values of the features are shown. Note that the classifier uses z-scale standardized features for placing uniform importance to features initially.

**Feature**	**pCR median (IQR)**	**nCR median (IQR)**	**P values adjusted**
Difference pre-contrast Mean	-0.10 (-0.44, 0.44)	0.30 (-0.03, 0.785)	**< 0.001**
Post-NAC post1 Gabor (90, 1.414) energy	2186 (1418, 2885)	2488 (692, 4097)	0.919
Post-NAC pre-contrast Gabor (0, 1.414) contrast	526 (322, 692)	467 (119, 752)	0.270
Pre-NAC pre-contrast Gabor (0, 1.414) homogeneity	4.3 (3.4, 5.3)	4.6 (3.4, 5.9)	0.550
Difference pre-contrast homogeneity	27.0 (11.0, 40.0)	14.9 (2.6, 31.4)	**0.004**
Post-NAC post1 homogeneity	2091 (1389, 2720)	2325 (676, 3892)	0.92
Post-NAC post2 Gabor (0, 1.414) correlation	62.0 (47.0, 74.0)	54.0 (41.0, 70.0)	0.082

Pre-NAC intra-tumor cluster entropy	1.5 (1.4, 1.6)	1.6 (1.5, 1.7)	0.150
Difference pre-contrast Gabor (0, 1.414) entropy	0.28 (-0.17, 0.78)	0.31 (-0.24, 0.74)	0.680
Difference post2 skewness	6.2 (3.2, 11.6)	3.5 (0.30, 8.20)	**0.0025**
Difference post1 Gabor (0, 1.1414) energy	0.09 (-0.22, 0.58)	0.47 (0.04, 0.88)	**0.0025**
Difference post1 mean	6.1 (3.2, 11.6)	3.5 (0.3, 8.2)	**0.003**

Supplemental Table 4: Performance of the RFE-RF classifier trained using Radiomics only model without mean MRI intensity values for predicting a pCR. P values are derived from comparison of the ROC curves computed for the cross-validation and test sets and comparison against Radiomics model (Model 1).

	**Radiomics without mean MRI intensity**	
	Training	***Testing***
AUROC 95% CI	0.71 (0.64, 0.79)	0.78 (0.62, 0.94)
Sensitivity or TPR (no-pCR)	0.73 (0.66, 0.80)	0.79 (0.64, 0.90)
Specificity or TNR (pCR)	0.61 (0.47, 0.73)	0.69 (0.39, 0.91)
PPV	0.83 (0.76, 0.89)	0.89 (0.75, 0.97)
NPV	0.47 (0.36, 0.58)	0.50 (0.42, 0.65)
	P-Value = 0.5	
Comparison against Model 1	P-Value = 1.0	P-Value = 0.4

Supplemental Table 5: Median and inter-quartile range (IQR) between pCR and nCR among all the features selected as relevant by RFE-RF classifier for the Radiomics only Model3 classifier where mean features were removed. Adjusted P values are shown. Actual values of the features are shown. Note that the classifier uses z-scale standardized features for placing uniform importance to features initially.

**Feature**	**pCR median (IQR)**	**nCR median (IQR)**	**P values adjusted**
Post-NAC post1 homogeneity	2091 (1389, 2720)	2325 (676, 3892)	0.985
Post-NAC pre-contrast Gabor (0, 1.414) energy	1589 (919, 1965)	1521 (419, 2288)	0.443
Post-NAC pre-contrast Gabor (0, 1.414) contrast	526 (322, 694)	467 (119, 752)	0.290
Difference pre-contrast homogeneity	27.0 (11.0, 40.0)	14.9 (2.6, 31.4)	**0.009**
Difference pre-contrast contrast	6.1 (2.8, 11.4)	3.5 (0.34, 8.47)	**0.012**
Post-NAC pre-contrast Gabor (90, 1.414) entropy	1944 (1250, 2439)	2036 (599, 3541)	0.980
Difference post1 Gabor (0, 1.1414) energy	0.09 (-0.22, 0.58)	0.47 (0.04, 0.88)	**0.007**
Difference post1 kurtosis	22.3 (4.6, 37.7)	15.0 (0.0, 30.0)	0.140
Difference pre-contrast Gabor (90, 1.414) correlation	-367 (-1117, 94)	-121 (-683, 104)	0.110
Difference post1 Gabor (90, 1.414) mean	-0.92 (-17.8, 13.9)	2.3 (-9.4, 15.6)	0.180
Pre-NAC post2 homogeneity	6.8 (5.0, 9.7)	6.5 (4.3, 9.6)	0.370
