# Supplementary material for: A machine learning model that classifies breast cancer pathologic complete response on MRI post-neoadjuvant chemotherapy
Source: Breast Cancer Res. 2020 May 28;22:57. doi: 10.1186/s13058-020-01291-w (PMC7254668; doi:10.1186/s13058-020-01291-w)
Supplement: Supplementary file 3 — Additional file 3. Supplemental Figure. [file 13058_2020_1291_MOESM3_ESM.pdf]

ADDITIONAL FILE 3

Supplemental Figure: Radiomic features associated with a pCR for the Radiomics only Model 1 classifier (A-I) and the radiomics with molecular subtype classifier Model 2 (J). Only features that were significantly associated with a pCR after adjustment for multiple comparisons are shown for each classifier category. The P-values correspond to the values produced after adjustment for multiple comparisons.

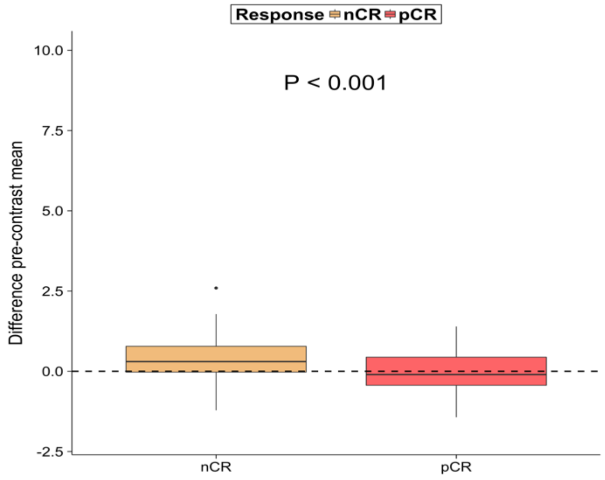

(A) Difference pre-contrast mean

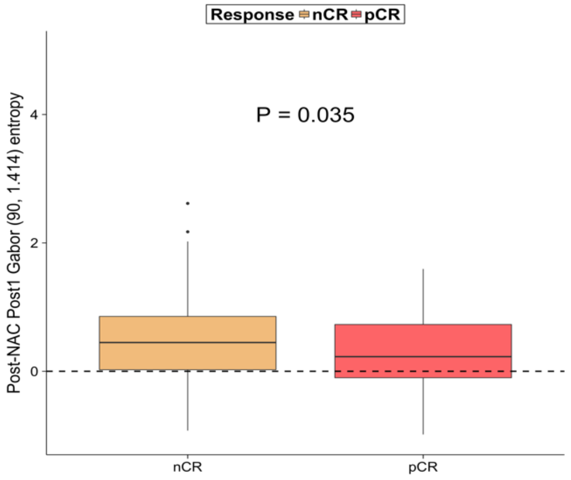

(B) Post-NAC Post1 Gabor (90, 1.414) entropy

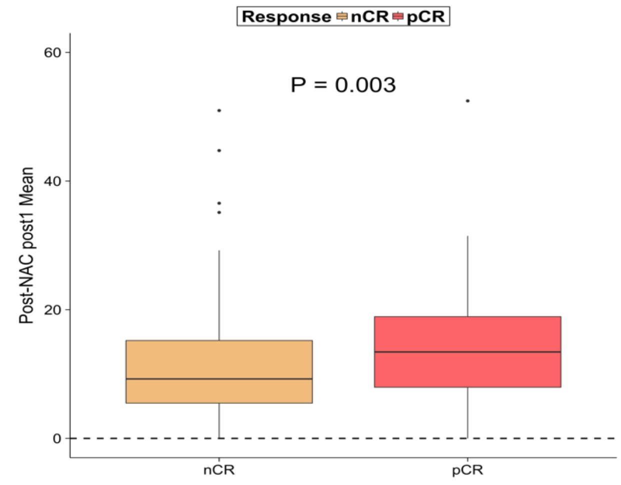

(C) Post-NAC post1 mean

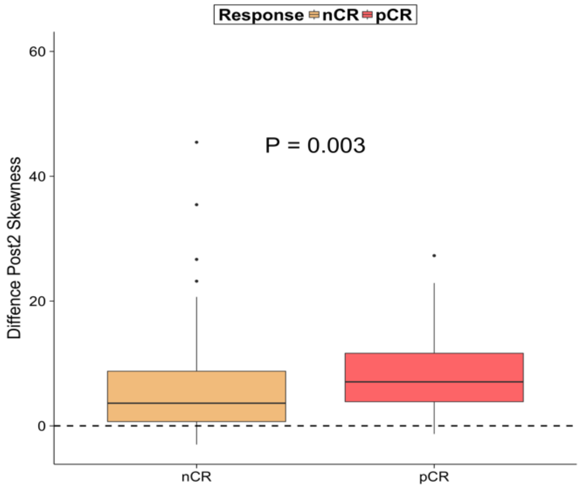

(D) Difference post2 skewness

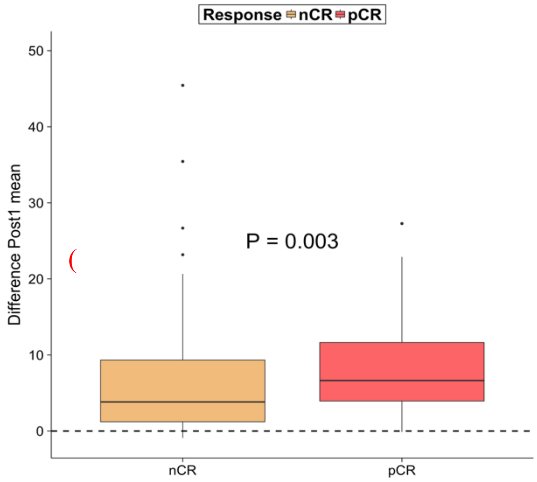

(E) Difference post1 mean

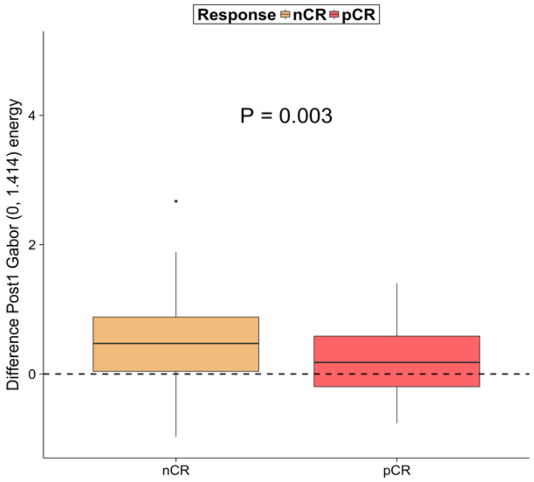

(F) Difference post1 Gabor (0, 1.414) energy

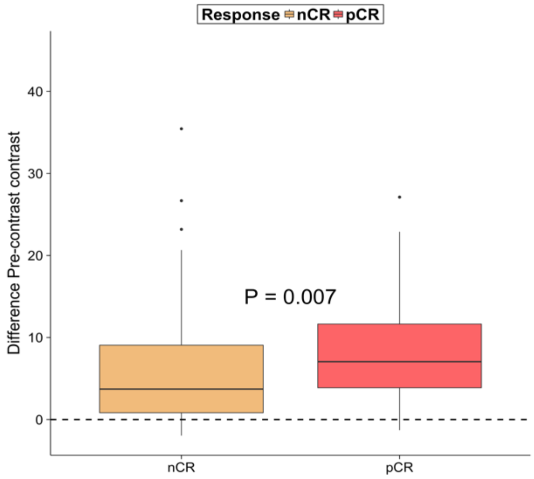

(G) Difference pre-contrast contrast

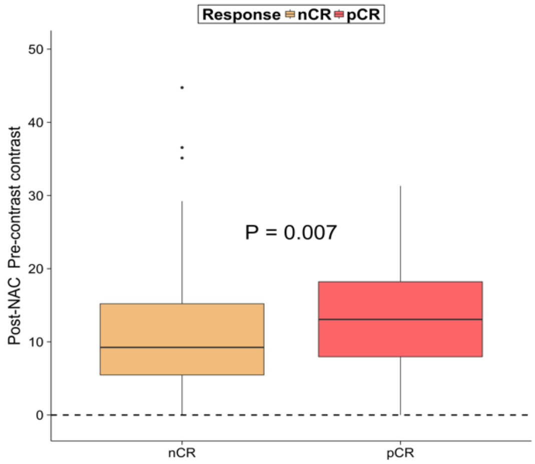

(H) Post-NAC pre-contrast contrast

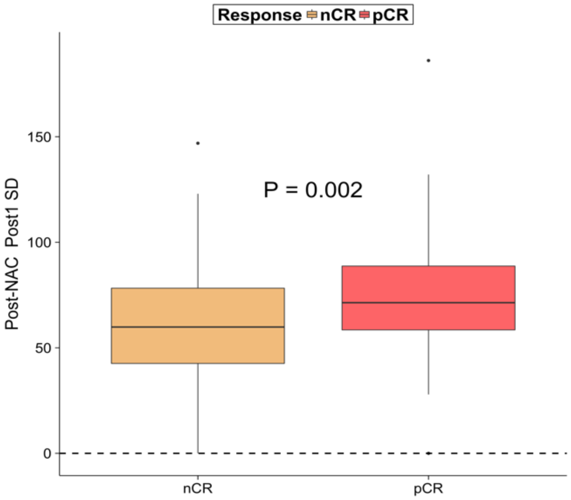

(I) Post-NAC post1 SD

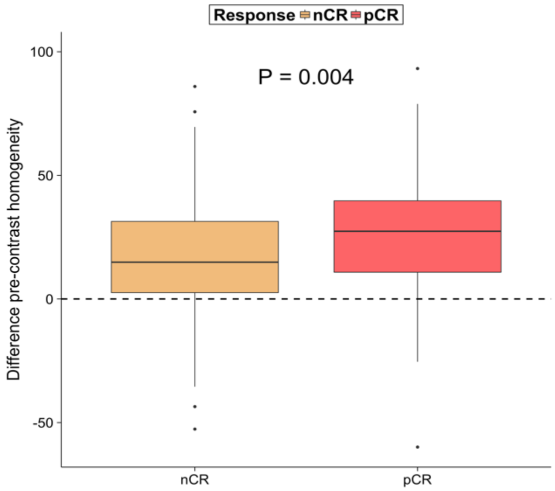

(J) Difference pre-contrast homogeneity
